# Bivariate Spatial Pattern between Smoking Prevalence and Lung Cancer Screening in US Counties

**DOI:** 10.3390/ijerph17103383

**Published:** 2020-05-13

**Authors:** Bian Liu, Jeremy Sze, Lihua Li, Katherine A. Ornstein, Emanuela Taioli

**Affiliations:** 1 Department of Population Health Science and Policy, Icahn School of Medicine at Mount Sinai, New York, NY 10029, USA; Lihua.Li@mountsinai.org (L.L.); emanuela.taioli@mountsinai.org (E.T.); 2 Institute for Translational Epidemiology, Icahn School of Medicine at Mount Sinai, New York, NY 10029, USA; katherine.ornstein@mssm.edu; 3 Department of Population Health, Division of Health & Behavior, Section on Health Choice, Policy & Evaluation, New York University School of Medicine, New York, NY 10016, USA; jeremy.sze@outlook.com; 4 Department of Geriatrics and Palliative Medicine, Icahn School of Medicine at Mount Sinai, New York, NY 10029, USA

**Keywords:** low-dose computed tomography, cigarette smoke exposure, health disparities, medicare, cancer screening

## Abstract

**Objectives:** Lung cancer screening (LCS) with low-dose computed tomography (LDCT) has been a reimbursable preventive service covered by Medicare since 2015. Geographic disparities in the access to LDCT providers may contribute to the low uptake of LCS. We evaluated LDCT service availability for older adults in the United States (US) based on Medicare claims data and explored its ecological correlation with smoking prevalence. **Materials and Methods:** We identified providers who provided at least 11 LDCT services in 2016 using the Medicare Provider Utilization and Payment Data: Physician and Other Supplier Public Use File. We constructed a 30-mile Euclidian distance buffer around each provider’s location to estimate individual LDCT coverage areas. We then mapped the county-level density of LDCT providers and the county-level prevalence of current daily cigarette smoking in a bivariate choropleth map. **Results:** Approximately 1/5 of census tracts had no LDCT providers within 30 miles and 46% of counties had no LDCT services. At the county level, the median LDCT density was 0.5 (interquartile range (IQR): 0–5.3) providers per 1000 Medicare fee-for-service beneficiaries, and cigarette smoking prevalence was 17.5% (IQR: 15.2–19.8%). High LDCT service availability was most concentrated in the northeast US, revealing a misalignment with areas of high current smoking prevalence, which tended to be in the central and southern US. **Conclusions:** Our maps highlight areas in need for enhanced workforce and capacity building aimed at reducing disparities in the access and utilization of LDCT services among older adults in the US.

## 1. Introduction

Lung cancer is the leading cause of cancer deaths in the United States (US), and it is also the second most common cancer (excluding skin cancer) in both men and women [1,2,3,4]. Treatment options such as surgery are the standard of care recommended for stages I-IIIA of non-small cell lung cancer, alone or in conjunction with radiation and/or chemotherapy (for more advanced stages) [5], while limited therapies are available for patients with late stage lung cancer. Thus, early detection is an important secondary prevention measure to reduce lung cancer mortality. Understanding the utilization pattern of cancer screening tests across different geographic regions is critical for planning cancer prevention and control strategies aimed at increasing screening uptake and reducing cancer disparities.

Adoption of annual lung cancer screening (LCS) with low-dose computed tomography (LDCT), which was recommended by the United States Preventive Services Task Force (USPSTF) in 2013, is a vital strategy for early lung cancer detection [6,7]. In 2015, the Centers for Medicare and Medicaid Services (CMS) endorsed similar guidelines by providing medical reimbursement for LCS with LDCT among eligible persons [8,9]. Similar to the USPSTF recommendation, the annual LCS under the CMS rule applies to asymptomatic adults aged 55–77 years (USPSTF recommends for age 55–80 years) with tobacco smoking history of at least 30 pack-years, which include both current smokers and former smokers who have quit smoking within the last 15 years [7,8,9].

Despite these recommendations and reimbursement policy changes, the overall uptake of LCS remains low among the general US population and among fee-for-service (FFS) Medicare beneficiaries [10,11,12]. One of the contributing factors for the slow adoption of LCS, as shown by our team and others, is the geographic disparities of the availability of LDCT screening facilities [13,14,15,16,17]. In the current study, we aim to examine the geographic accessibility of LDCT service and explore its bivariate relationship with smoking prevalence. We present census-tract- and county-level data, an improvement of previous studies based on either state- or national-level aggregated data (or both). Counties with high smoking prevalence and low LDCT services may indicate potential opportunities for enhanced LCS interventions.

## 2. Methods

LDCT providers and utilizations: Data on LDCT utilization were obtained from the Medicare Provider Utilization and Payment Data: Physician and Other Supplier Public Use File for the calendar year 2016 [18]. The provider PUF includes summaries of services and procedures provided by physicians and other healthcare professionals to Medicare FFS beneficiaries. Note that only data from FFS Medicare beneficiaries are available, which represent about 70% of all Medicare beneficiaries and 83% of the 47.8 million people aged 65 years and older in the US. Given that 70% of lung cancer is diagnosed among people aged 65 years and older [19], FFS Medicare data are appropriate for the study. Data for providers who serviced less than 11 FFS beneficiaries were suppressed from the publicly released data for privacy concerns. We identified LDCT performed for LCS based on the Healthcare Common Procedure Coding System (HCPCS) code G0297, and summed all LDCT services utilized for individual providers based on their unique national provider identifier (NPI). We geocoded the address of each provider to obtain the latitude and longitude using the Texas A&M Geocoding Services.

Medicare population by census tracts and counties: We utilized the 2012–2016 American Community Survey (ACS) 5-year estimates to identify the number of Medicare beneficiaries aged 65 years and over in each census tract, excluding 611 census tracts with zero Medicare beneficiaries [20]. We obtained Medicare FFS population in each county from the Medicare Fee-for-Service Enrollment Public Use File for 2016 [21]. Note that the denominator population, while representative of Medicare population, differs from those screening eligible individuals defined by USPSTF and CMS.

Current smoking prevalence by counties: Current smoking prevalence estimates are only publicly available at the county level, not at the census tract level, for the entire US (excluding US territories). We obtained the county-level age-standardized current cigarettes smoking prevalence from a previously published study by Dwyer-Lindgren et al. [22], in which authors applied a validated small-area-estimate approach to obtain two types of current smoking prevalence based on the 1996–2012 survey data from the Behavioral Risk Factor Surveillance System (BRFSS), a national population-based survey for adults (≥18 years) [23]. In BRFSS, current smokers were identified based on combined responses to the questions “Have you smoked at least 100 cigarettes in your entire life?” and “Do you now smoke cigarettes every day, some days or not at all?” The total cigarette smoking prevalence was from both nondaily and daily smoker combined data, while daily cigarette smoking prevalence was from daily smoker data only. In this study, we used daily cigarette smoking prevalence (referred to as “current smoking prevalence” hereafter), since daily smokers may better meet the smoking intensity eligibility (30 pack-year) for LCS than nondaily smokers. We used data for the calendar year 2012, which were the latest available year to the LDCT utilization data and the most up-to-date publicly available county-level data.

LDCT provider density by census tracts and counties: We constructed a 30-mile Euclidian distance buffer around each provider’s location, as in previous studies [24,25], to identify the coverage areas of the providers. A census tract was considered to be served by a provider if more than 51% of its area was within the 30-mile radius of the provider’s geolocation. We summed the number of providers from all providers within a census tract, which was subsequently aggregated to the county level. We calculated the LDCT provider density at the tract and county level, respectively, using the summed numbers of LDCT providers as the numerator and the Medicare population at tract and county levels, respectively, as the denominator. The LDCT provider service areas at the census tract level were calculated using the 2016 census tract shapefile from the National Historical Geographic Information System from IPUMS [20]. The analysis was conducted with Python 3.7.3 using the following libraries Geopandas 0.5.1 and Pysal 2.1.0 in JupyterLab [26,27,28,29,30,31,32,33,34,35].

Geospatial analysis: To match the county-level current smoking prevalence, we mainly focused on county-level data in the current study and presented the tract-level data as supplemental information (Figure S1). We visualized the provider density and smoking prevalence with individual choropleth maps using the 2016 ACS county shapefile from the US Census Bureau. In addition, we produced a bivariate choropleth map and a scatter plot to show the overlaps between smoking prevalence and provider density. To that end, we calculated the percentiles of individual variables, and generated the corresponding 3-level variable to represent low (<34th percentile), medium (≥34th and <67th percentiles), and high (≥67th percentile) ranking categories, respectively. The analysis and mapping were conducted with R (V 3.5.0) in RStudio (V 1.1.453) using the following libraries: tidycensus, sf, dplyr, pals, classInt, tmap, latticeExtra, ggpubr, and grid [36,37,38,39,40,41,42,43,44,45].

## 3. Results

**Figure 1:**
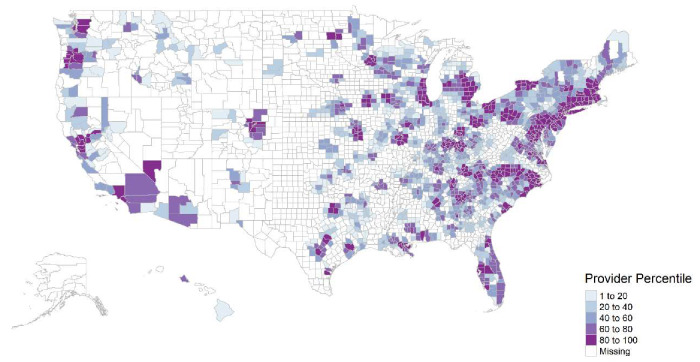
County-level density of providers, who provided low-dose computed tomography (LDCT) services for lung cancer screening, per 1000 Medicare fee-for-service beneficiaries, 2016.

We found 50915 LDCT utilizations billed by 1860 providers from 50 US states and the District of Columbia in 2016. The average LDCT service per individual provider was 27 ± 25, with a median of 19 and a minimum and maximum of 11–302. Approximately 21.8% (16,082/73,665) of census tracts had no LDCT providers within 30 miles of their locations. Approximately 46.5% (1461/3142) of counties had no LDCT services, shown as missing in Figure 1. The median and interquartile range (IQR) of Medicare FFS population at the county level were 3938 (IQR: 1856–9617). The median LDCT provider density was 0.5 (IQR: 0–5.3) providers per 1000 Medicare FFS beneficiaries at the county level. Among counties that had LDCT services (*n* = 1681), the median LDCT provider density was 4.6 (IQR: 0.03–12.1) providers per 1000 Medicare FFS beneficiaries. The median prevalence of current smoking by county was 17.5% (IQR: 15.2–19.8%).

Table 1 lists the top- and bottom-ranked ten counties for the LDCT provider density and current smoking prevalence. The ten counties with top-ten LDCT provider density, all of which had more than 129 providers per 1000 Medicare FFS beneficiaries, were Suffolk, Middlesex, Norfolk, and Essex counties of Massachusetts; Bronx, Queens, and Kings counties of New York; Wayne county of Michigan; Philadelphia county in Pennsylvania; and Hudson county in New Jersey. The current daily cigarette smoking prevalence for these ten counties ranged from 9.9 to 18.2%. Counties with top-ten prevalence of current smoking were Clay, Knox, Elliott, Leslie, Lee, and Jackson counties in Kentucky; Northwest Arctic Borough in Alaska; and McDowell, Roane, and Boone counties in West Virginia. Four out of these ten counties did not have LDCT services, and the LDCT provider density in the remaining six counties ranged from 0.4 to 7.6 providers per 1000 Medicare FFS beneficiaries. Among the ten counties with bottom-ten LDCT provider density, the prevalence of current smoking varied between 9.6% and 24.1%. Four out of the ten counties with the lowest current smoking prevalence had no LDCT services, all of which were in Utah; the LDCT provider density in the remaining six counties ranged from 14.2 to 68.6 providers per 1000 Medicare FFS beneficiaries. Large variations in both LDCT provider density and smoking prevalence exist across counties throughout the US, as well as across counties within the same state. For example, Lackawanna County and Philadelphia County of Pennsylvania were within the bottom- and top-ten counties of LDCT provider density, respectively.

**Figure 2:**
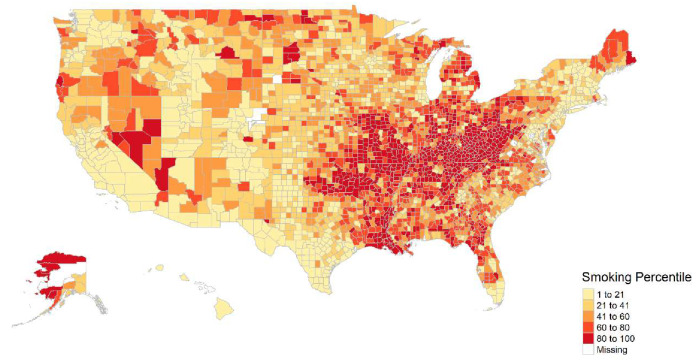
County-level prevalence of age-standardized current daily cigarette smoking, 2012.

**Figure 3:**
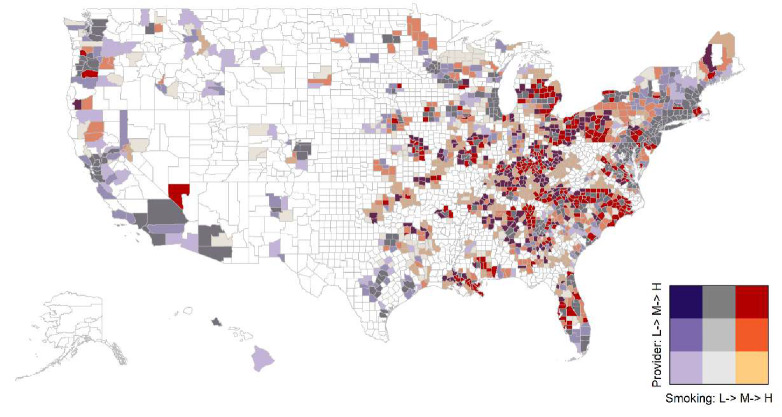
Bivariate choropleth map between current daily cigarette smoking prevalence and the density of providers, who provided low-dose computed tomography (LDCT) services for lung cancer screening, per 1000 Medicare fee-for-service beneficiaries. Approximately 46.5% (1461/3142) of counties had no LDCT services, shown in white color. The color scheme shows the nine combinations of the LDCT provider density and smoking prevalence patterns by tertiles: low–low, low–medium, low–high, medium–low, medium–medium, medium–high, high–low, high–medium, and high–high.

**Figure 4:**
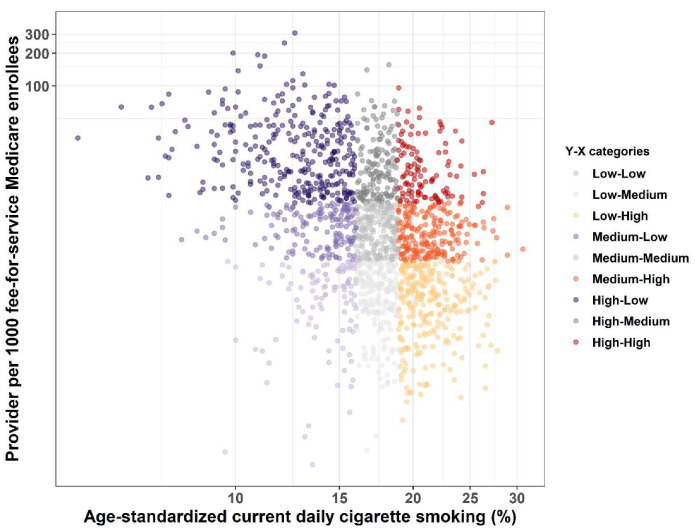
Scattered plot between current daily cigarette smoking prevalence and the density of providers, who provided low-dose computed tomography (LDCT) services for lung cancer screening, per 1000 Medicare fee-for-service beneficiaries. The color scheme shows the nine combinations of the LDCT provider density and smoking prevalence patterns by tertiles: low–low, low–medium, low–high, medium–low, medium–medium, medium–high, high–low, high–medium, and high–high.

Both the LDCT provider density and current smoking prevalence varied substantially across US counties. Higher LDCT provider density generally concentrated in the northeast than south and west US, as shown in Figure 1, while higher smoking prevalence occurred in the south and central US, as shown in Figure 2. The mismatch between current smoking prevalence and the LDCT provider density was further evidenced by mapping both variables in a bivariate choropleth map shown in Figure 3 and in a scattered plot shown in Figure 4, depicting the nine combinations of the LDCT provider density and smoking prevalence patterns: low–low, low–medium, low–high, medium–low, medium–medium, medium–high, high–low, high–medium, and high–high. Again, regions in the south where high smoking prevalence occurred tended to have low or no LDCT services.

## 4. Discussion

We present estimates of LDCT utilization for lung cancer screening in the US based on the 2016 FFS Medicare claims data, one year after Medicare’s reimbursement began in 2015. We found substantial spatial variations in the coverage areas of the providers who furnished LDCT services to Medicare FFS beneficiaries. These variations of LDCT accessibility do not align with known geographic distributions of current smoking prevalence, which tend to be in the central and southern US, where high lung cancer incidence and mortality are also found [1,2,3,4,13,14,15,16,17]. Notably, counties in southern states, such as Kentucky and West Virginia, tend to simultaneously rank high in current smoking prevalence and rank low in LDCT provider density. In contrast, counties in northeastern states, such as Massachusetts, New York, and New Jersey, which have below median smoking prevalence, tend to rank high in LDCT provider density. Counties in western states, such as Utah and parts of California, tend to rank low in smoking prevalence and low in LDCT providers. The findings of these regional geographic variations were consistent with results from previous studies [13,14,15,16,17]. Possible explanations of the observed mismatch between smoking prevalence and the LDCT provider coverage include discrepancies in the underlying population characteristics, individual and contextual socioeconomic conditions, as well as the distributions of healthcare resources and population densities in urban and rural areas. These variations have been shown to affect the distribution of LDCT facilities, adoption of screening technologies, and uptake of LCS, and have been observed for other cancer screening services [46,47,48,49,50,51].

We built upon findings from previous studies on LDCT availability and accessibility that used aggregated data at the state or national level by examining data at the census tract and county level. With improved refinement in the geographic resolution, we were able to demonstrate variations across all counties in the nation and within the same state, which may not be captured with state-level data. These county-level variations provided a more granulated and likely realistic assessment of the LDCT service availability and accessibility. Our county-level estimates can be applied to inform future federal, state, and local initiatives aimed at improving LCS uptake. For example, counties with high smoking prevalence and low LDCT utilizations may indicate potential opportunities for localities to increase access to LCS interventions.

There are a few limitations in our study. First, our findings are representative of the Medicare FFS population, which differs from the population that are screening eligible defined by USPSTF and CMS. Unfortunately, detailed smoking status and smoking intensity are not available to accurately estimate LCS-eligible population, which suggests that our provider density estimates are likely to be underestimated. Second, our data were based on FFS Medicare beneficiaries which, while represent 83% of the US population aged 65 years and older, may not be generalizable to older adults enrolled in Medicare Advantage, commercial insurances, or the Veterans Health Administration healthcare system. Third, while, to the best of our knowledge, the 2012 smoking prevalence data we include are the most up-to-date, age-adjusted estimates available at the county level, the prevalence may have changed. Finally, the use of a fixed 30-mile radius to define the LDCT service area for all providers, while a reasonable average, may underestimate the coverage area for providers residing in rural areas. Other approaches, such as using adaptive radii, cumulative distribution functions, or network buffers, are warranted in future studies to improve estimates [52,53].

## 5. Conclusions

Our results identified potential geographic disparities in the access and utilization of LDCT services among older adults in the US. Increased workforce and capacities for lung cancer screening in areas with low distribution of LDCT providers, especially in target areas where smoking prevalence is high, is crucial to ensure the continued adoption of LDCT and the success of lung cancer screening efforts.

## Figures and Tables

**Table 1:** Counties with top- and bottom-ranked current daily cigarette smoking prevalence, and density of providers, who provided low-dose computed tomography (LDCT) services for lung cancer screening, per 1000 Medicare fee-for-service (FFS) beneficiaries.

Variables	LDCT Provider Density (Provider per 1000 Medicare FFS Beneficiaries)	Current Daily Cigarette Smoking Prevalence (%)	County
**Top-ten LDCT provider density**	129.7	13.0	Essex County, Massachusetts
	138.1	10.1	Hudson County, New Jersey
	140.3	16.7	Philadelphia County, Pennsylvania
	153.4	11.0	Norfolk County, Massachusetts
	156.9	18.2	Wayne County, Michigan
	188.5	11.2	Middlesex County, Massachusetts
	194.3	10.9	Kings County, New York
	201.4	9.9	Queens County, New York
	249.2	12.1	Bronx County, New York
	308.8	12.6	Suffolk County, Massachusetts
**Bottom-ten provider density**	0.032	13.5	Yakima County, Washington
	0.043	9.6	Imperial County, California
	0.044	16.7	Clallam County, Washington
	0.059	13.1	Tippecanoe County, Indiana
	0.060	17.3	Lackawanna County, Pennsylvania
	0.074	13.3	Monroe County, Florida
	0.084	19.2	Polk County, Texas
	0.098	15.6	Okaloosa County, Florida
	0.123	19.5	Knox County, Illinois
	0.134	24.1	Sequoyah County, Oklahoma
**Top-ten smoking prevalence**	1.6	27.6	Boone County, West Virginia
	4.0	27.7	Jackson County, Kentucky
	not available	27.8	Lee County, Kentucky
	not available	27.8	Leslie County, Kentucky
	0.4	27.8	Roane County, West Virginia
	not available	28.0	McDowell County, West Virginia
	7.6	28.9	Elliott County, Kentucky
	2.8	28.9	Knox County, Kentucky
	not available	29.8	Northwest Arctic Borough, Alaska
	3.1	30.7	Clay County, Kentucky
**Bottom-ten smoking prevalence**	not available	5.7	Utah County, Utah
	64.1	6.4	Arlington County, Virginia
	not available	7.0	Summit County, Utah
	not available	7.0	Wasatch County, Utah
	14.2	7.1	Santa Clara County, California
	63.9	7.2	Montgomery County, Maryland
	not available	7.2	Davis County, Utah
	14.4	7.4	San Mateo County, California
	33.2	7.4	Loudoun County, Virginia
	68.6	7.5	Howard County, Maryland

## Supplementary Materials

The following are available online at https://www.mdpi.com/1660-4601/17/10/3383/s1, Figure S1: Census-tract-level density of providers, who provided low-dose computed tomography (LDCT) services for lung cancer screening, per 100 Medicare fee-for-service beneficiaries, 2016.
