# Supplementary material for: Bivariate Spatial Pattern between Smoking Prevalence and Lung Cancer Screening in US Counties
Source: Int J Environ Res Public Health. 2020 May 13;17(10):3383. doi: 10.3390/ijerph17103383 (PMC7277441; doi:10.3390/ijerph17103383)
Supplement: Supplementary file 1 [file ijerph-17-03383-s001.pdf]

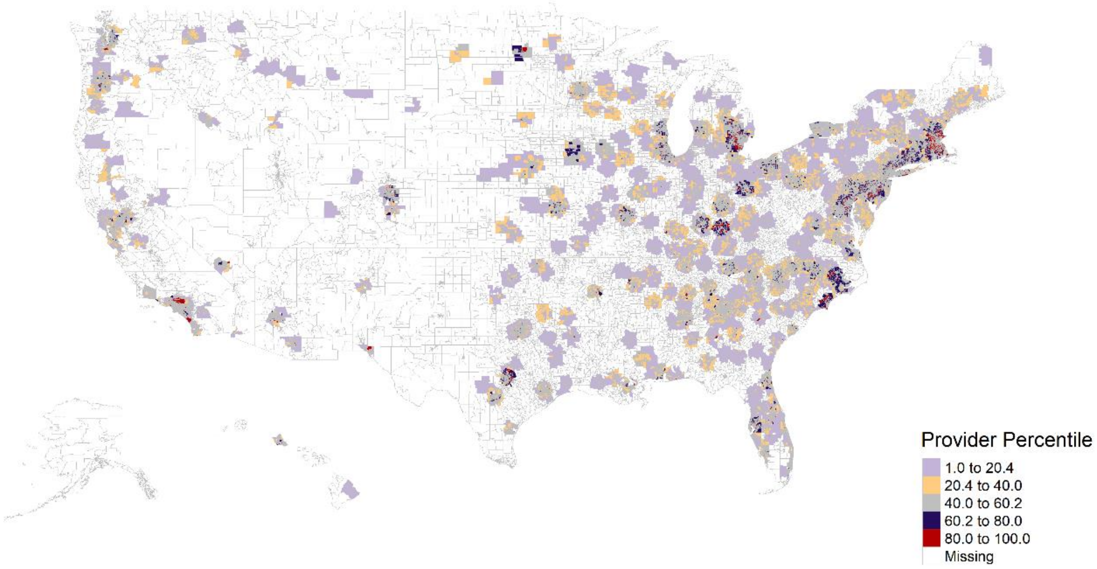

Figure 1. Census-tract-level density of providers, who provided low-dose computed tomography (LDCT) services for lung cancer screening, per 100 Medicare fee-for-service beneficiaries, 2016.
